# Knowledge Structure and Emerging Trends of Telerehabilitation in Recent 20 Years: A Bibliometric Analysis *via* CiteSpace

**DOI:** 10.3389/fpubh.2022.904855

**Published:** 2022-06-20

**Authors:** Jiaqi Zheng, Meijin Hou, Lu Liu, Xiangbin Wang

**Affiliations:** ^1^College of Rehabilitation Medicine, Fujian University of Traditional Chinese Medicine, Fuzhou, China; ^2^Key Laboratory of Orthopedics & Traumatology of Traditional Chinese Medicine and Rehabilitation Ministry of Education, Fujian University of Traditional Chinese Medicine, Fuzhou, China; ^3^National-Local Joint Engineering Research Center of Rehabilitation Medicine Technology, Fuzhou, China

**Keywords:** telerehabilitation, CiteSpace, bibliometric analysis, Web of Science, co-citation

## Abstract

**Purpose:**

Telerehabilitation, as an effective means of treatment, is not inferior to traditional rehabilitation, and solves the problem of many patients who do not have access to hospital-based training due to costs and distance. So far, the knowledge structure of the global use of telerehabilitation has not been formed. This study aimed to demonstrate the state of emerging trends and frontiers concerning the studies of telerehabilitation through bibliometric software.

**Methods:**

Literature about telerehabilitation from 2000 to 2021 was retrieved from the Web of Science Core Collection. We used CiteSpace 5.8.R3 to analyze the publication years, journals/cited journals, countries, institutions, authors/cited authors, references, and keywords. Based on the analysis results, we plotted the co-citation map to more intuitively observe the research hotspots and knowledge structure.

**Results:**

A total of 1,986 records were obtained. The number of annual publications gradually increased over the investigated period. The largest increase occurred between 2019 and 2020. J TELEMED TELECARE was the most prolific and the most cited journal. The United States was the most influential country, with the highest number of publications and centrality. The University of Queensland was the most productive institution. The author Tousignant M ranked the highest in the number of publications and Russell TG ranked the first in the cited authors. Respectively, the articles published by Cottrell MA and Russell TG ranked the first in the frequency and centrality of cited references. The four hot topics in telerehabilitation were “care”,“stroke”, “telemedicine” and “exercise”. The keyword “stroke” showed the strongest citation burst. The two frontier keywords were “physical therapy” and “participation”. The keywords were clustered to form 21 labels.

**Conclusion:**

This study uses visualization software CiteSpace to provide the current status and trends in clinical research of telerehabilitation over the past 20 years, which may help researchers identify new perspectives concerning potential collaborators and cooperative institutions, hot topics, and research frontiers in the research field. Bibliometric analysis of telerehabilitation supplements and improves the knowledge field of telemedicine from the concept of rehabilitation medicine and provides new insights into therapists during the COVID-19 pandemic.

## Introduction

During the past two decades, the growing availability of communication technologies (ICTs) has created the opportunity to provide technology-based health care in the hospital or after discharge (1). This way, broadly referred to as telemedicine, may ensure the provision of approachable, cost-effective, and particular health care services in disparate times and areas. ICTs have also shown incredible promise in rehabilitation, encouraging the birth of a fresh branch of telemedicine, known as telerehabilitation. Telerehabilitation is attracting considerable critical attention. Extensive research has shown that telerehabilitation is equal or more effective and less costly compared with traditional face-to-face rehabilitation in motor impairments, postoperative recovery, pulmonary function, cardiovascular disease, and other health-related problems (2–4). Especially during the COVID-19 pandemic, under the circumstance of impacting residents' travel and imposing social distance, telerehabilitation may seem to resemble a feasible alternative to face-to-face delivery of rehabilitation services during and after the enhanced quarantine period (5). Thus, telerehabilitation is an increasingly important area nowadays in society. However, few studies are concerned with the comprehensive knowledge structure of telerehabilitation.

Bibliometrics refers to the quantitative analysis of all knowledge carriers, mainly published literature by mathematical and statistical methods, which can be used to assess the impact of authors, institutions, countries, and keywords on the growth of specific fields according to the number of and citation frequency of publications (6). It helps find the trends and hotspots to form the knowledge structure (7). Gu et al. (8) used bibliometrics to explore the trends in the development of e-health and telemedicine research on the retrieved 3,085 papers from the Web of Science Core Collection in 1992–2017. Ahmed Waqas et al. presented an overview of scholarly work in the field of telemedicine based on CiteSpace in 2010–2019 (9). Thus, Telemedicine has become a hot topic and telerehabilitation research is a branch derived from it. No one visualizes the comprehensive knowledge structure, evolutionary path, and research hotspots of telerehabilitation from the perspective of bibliometrics. The above two articles were published before the COVID-19 outbreak. Since the COVID-19 outbreak in 2019, the demand for telemedicine, especially telerehabilitation, has been increasing, and many researchers have been involved in the study of telerehabilitation. Bibliometrics research on telerehabilitation can be used as a complement to telemedicine knowledge and provide new inspiration for rehabilitation medicine, to promote the further development of remote technology. CiteSpace has been a common tool for bibliometrics analysis, which can provide intuitive information and potential research directions for researchers intuitively. Therefore, this study aimed to use CiteSpace to analyze retrieved records from the Web of Science Core Collection in the past 20 years to provide a better understanding of the development trends and current research status of telerehabilitation for researchers and new people in this field, thereby, guiding future research.

## Materials and Methods

### Data Acquisition

The data of this study were collected from the Web of Science (WOS) including SCI-E, SSCI, A&HCI, CPCI-S, CPCI-SSH, ESCI, CCR-E, and IC in January 2022. The data search strategy was as follows: Topic=(“telerehabilitation”) OR Topic=(“remote rehabilitation”). The retrieval period time was from January 2000 to December 2021. If there were no restrictions on language and literature type, 2,166 records were generated in the first query (“telerehabilitation”) and 99 records in the second query (“remote rehabilitation”). Subsequently, we processed the retrieved records. The first step was to merge the records retrieved from the two search terms and remove the duplicates. The second step was to retain articles, proceedings papers, and reviews, which were formally published and had comprehensive research data, then removed the document type of letter, meeting abstract, editorial material, etc. Finally, a total of 1,986 records were obtained.

### CiteSpace

CiteSpace is a Java-based application, developed by Professor Chaomei Chen, which visualizes interrelationships between scientific articles according to their co-citation patterns (10). Visualization knowledge maps show networks as the commonly seen types of node-and-link diagrams. Nodes in different networks can represent different elements, such as country, author, institution, and keyword. The size of the node, which generally indicates the frequency of citation or appearance, and the different colors of nodes show the different years. The warmer the color, the more recent the year, and the colder the color, the more distant the year. Links between nodes signify relationships of collaboration or cooccurrence or co-citation. The purple ring represents centrality. Nodes with high centrality (>0.1) are commonly regarded as pivotal points or turning points in a specific field. The version of this software is constantly being updated. In addition, CiteSpace provides two indicators, module value (Q value) and average contour value (S value), according to the network structure and clustering clarity, which can be used as the basis for judging the mapping effect. Generally speaking, Q value >0.3 means that the community structure is significant, while S value >0.5 means that the clustering is generally considered reasonable. The version used in this research was 5.8.R3 (64-bit). The parameters of CiteSpace were as follows: time slicing (2000–2021), years per slice (1), term source (all selection), node type (choose one at a time), selection criteria (top 50 objects), pruning (pruning sliced networks), and visualization (cluster view-static). We used CiteSpace to identify the time, frequencies, and centralities of the cooccurrence networks, which involved annual publications, journals, countries, institutions, authors, references, and keywords.

## Results

### Analysis of Annual Publications

**Figure 1:**
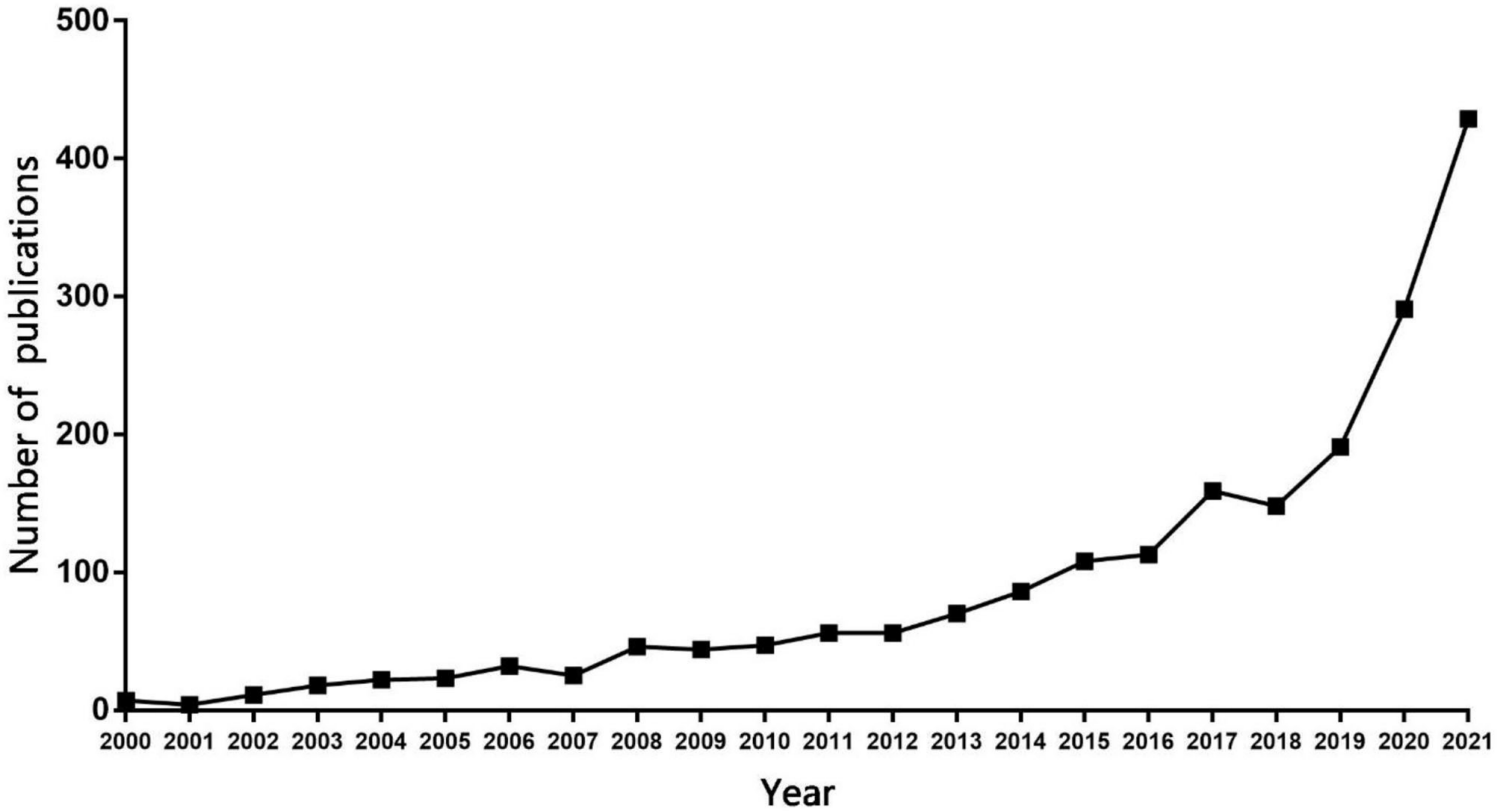
Annual number of published articles from 2000 to 2021.

In total, 1,986 records were included. The number of publications by years is shown in Figure 1. From the figure, we can see the number of publications increased with some fluctuations over 20 years. The number of publications was only 7 in 2000. There were fewer in 2001. It may support that the study and application of telerehabilitation started around the 21st century. Since 2002, the number had risen slowly during 5 years, and declined to 25 in 2007, and then went up again. In 2011 and 2012, the number was the same, 56. The period from 2013 to 2017 was a continued development period; the number exceeded 100 in 2015. Although it fell in 2018, there was substantial growth from 2019 to 2021. The publication outputs were 191 records in 2019. In 2021, the number of articles published was about four times that in 2019 with 429, probably because the impact of the COVID-19 pandemic caused more attention to telerehabilitation.

These results indicated that telerehabilitation, as a medical technology conceived in a new era, is receiving increased attention and more related research is being performed.

### Analysis of Journals and Cited Journals

The top five journals with the largest number of published telerehabilitation studies are listed in Table 1. Of the five journals, the top three are telemedicine-related journals. FRONT NEUROL and SENSORS show an emphasis on telerehabilitation. Except for INT J TELEREHABILITA, which did not have the most recent JCR journal citation report, the average IF of the remaining four journals was 4.325.

**Table 1:** Top five most productive journals related to telerehabilitation.

**Rank**	**Publications**	**Journal/IF^***a***^**
1	101	J Telemed Telecare/6.184
2	67	Int J Telerehbilita
3	47	Telemed e-Health/3.536
4	34	FRONT NEUROL/4.003
5	33	Sensors/3.576

*^a^ IF, impact factor; IF in category according to Journal Citation Reports (2021)*.

**Figure 2:**
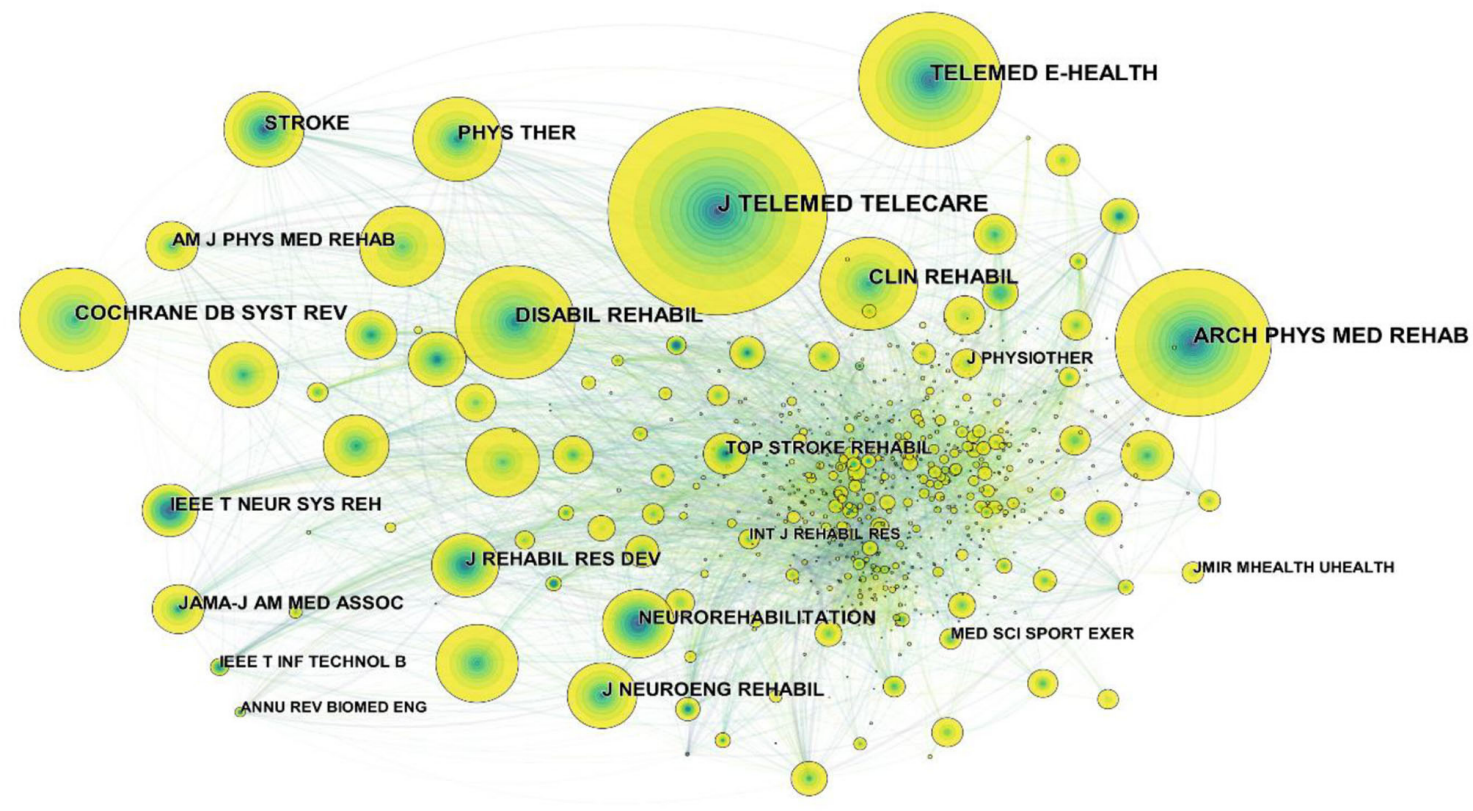
Map of cited journals for telerehabilitation from 2000 to 2021.

In addition, a cited journal map was generated by CiteSpace (Figure 2), resulting in 960 nodes and 7,601 links. The nodes in the map represented journals, and links between the nodes meant co-citation relationships. It can be seen from the figure that these nodes had no purple rings, indicating that the centrality of these journals was not high, the highest was 0.09 from ANNU REV BIOMED ENG. The top 10 cited journals related to telerehabilitation are shown in Table 2. We analyzed the bibliometric information of these ten journals (Table 3). Because the five selected bibliometrics depicted non-normal distribution, we reported the median, quartiles, and IQR. Most of the journals belonged to Q1, and more than half of the cited journals had an IF above 4.666. The median for the Eigenfactor was 0.0381 and for the CiteScore was 5.750. The median of SNIP and SJR was 1.280 and 1.710, respectively. The impact factor had a range between one and ten; the Eigenfactor score for most of the journals remained below 0.0615. The CiteScore for most journals remained between 3.9 and 7.1. The values of SNIP were primarily concentrated between 0.905 and 1.350. The SJR had a widespread between 0 and 2.5.

**Table 2:** Top 10 cited journals related to telerehabilitation.

**Rank**	**Frequency**	**Cited Journal**	**Rank**	**Frequency**	**Cited journal**
1	997	J TELEMED TELECARE	6	458	Clin Rehabil
2	727	ARCH PHYS MED REHAB	7	437	Phys Ther
3	669	TELEMED E-HEALTH	8	390	Stroke
4	556	DISABIL REHABIL	9	390	J Med Internet Res
5	490	COCHRANE DB SYST REV	10	381	Int J Telerehabilita

**Table 3:** Descriptive statistics of the bibliometrics from the top-10 cited journals.

**Bibliometrics**	**Median**	**25**	**75**	**IQR**
IF	4.666	3.030	6.617	3.587
Eigenfactor	0.0381	0.0065	0.0615	0.0550
CiteScore	5.750	3.900	7.100	3.200
SNIP	1.280	0.905	1.350	0.446
SJR	1.710	1.458	2.079	0.620

### Analysis of Countries

**Figure 3:**
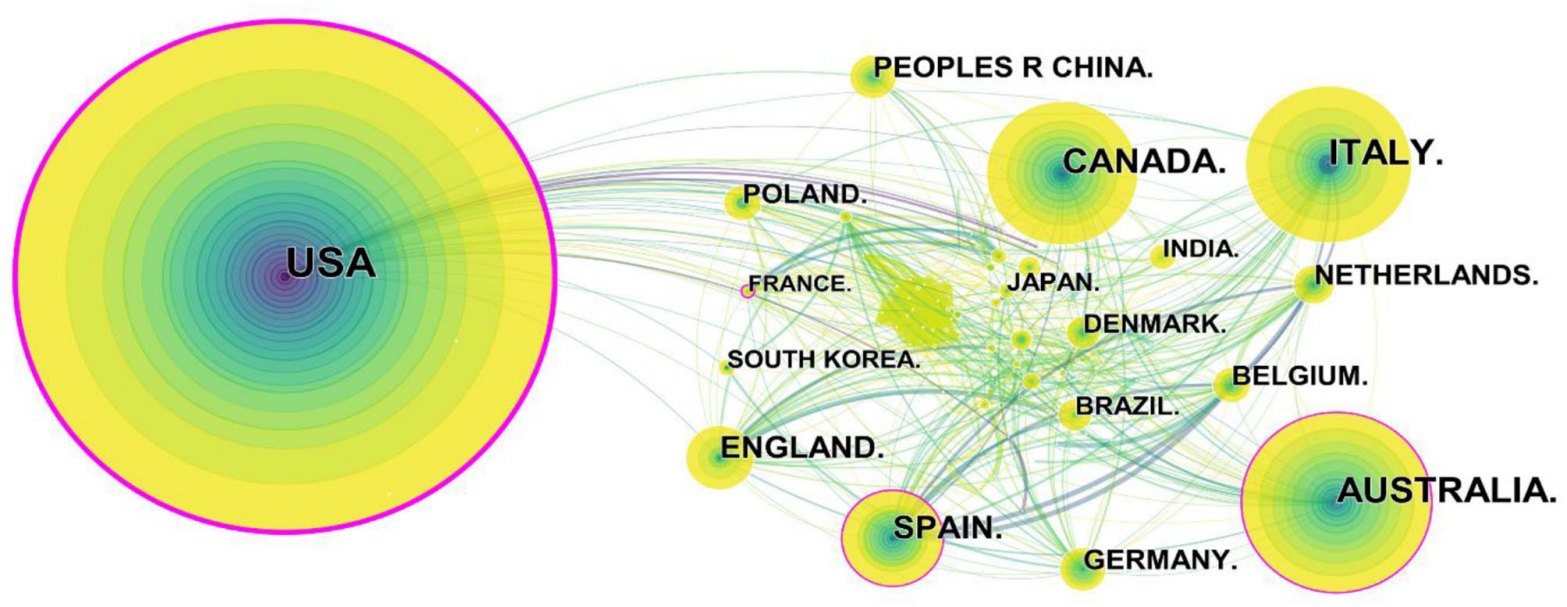
Map of countries researching telerehabilitation from 2000 to 2021.

We used CiteSpace to generate a country map, 142 nodes and 627 links were generated (Figure 3). Telerehabilitation was a concern in many countries. The included 1,986 articles were published by researchers in 142 countries. The top five countries of publications are displayed in Table 4. The United States was the main contributor, accounting for a third of the total articles (663), and it was published earlier than any other country. Australia and Italy were ranked in the second and third positions, respectively. Canada and Spain followed with more than 100 articles, while the rest of the country remained below. Except for the United States, the top five countries published their first articles on telerehabilitation around 2006. We can find that the top five countries are all developed countries with high economic and technological levels, which is conducive to the promotion and development of remote services. Additionally, China (including Taiwan) was the most productive country in Asia with 83 articles. China is the birthplace of traditional kung fu. The study applied the qigong exercise (Liu Zi Jue) under the guidance of remote video combined with acupuncture therapy to the treatment of severe COVID-19 patients and found that patients' symptoms, such as dyspnea and cough, were significantly improved, and their hospital stay was shortened (11).

**Table 4:** Top 5 countries in number of publications related to telerehabilitation.

**Rank**	**Publications**	**Countries**	**Year**
1	663	United States	2000
2	241	Australia	2007
3	213	Italy	2006
4	196	Canada	2006
5	135	Spain	2006

From Figure 3, the nodes represented the country, the purple ring indicated the centrality of literature, and the top five countries in terms of centrality were the United States (0.40), Spain (0.15), Australia (0.13), France (0.13), and Belgium (0.09). The United States had an advantage in terms of volume and importance. North America and Europe took the lead in conducting telerehabilitation research in the world. Telerehabilitation was gaining popularity in Asia after 2019, such as in Japan, India, China, and South Korea, which was reflected in the sharp rise in the number of articles.

### Distribution of Institutions

**Figure 4:**
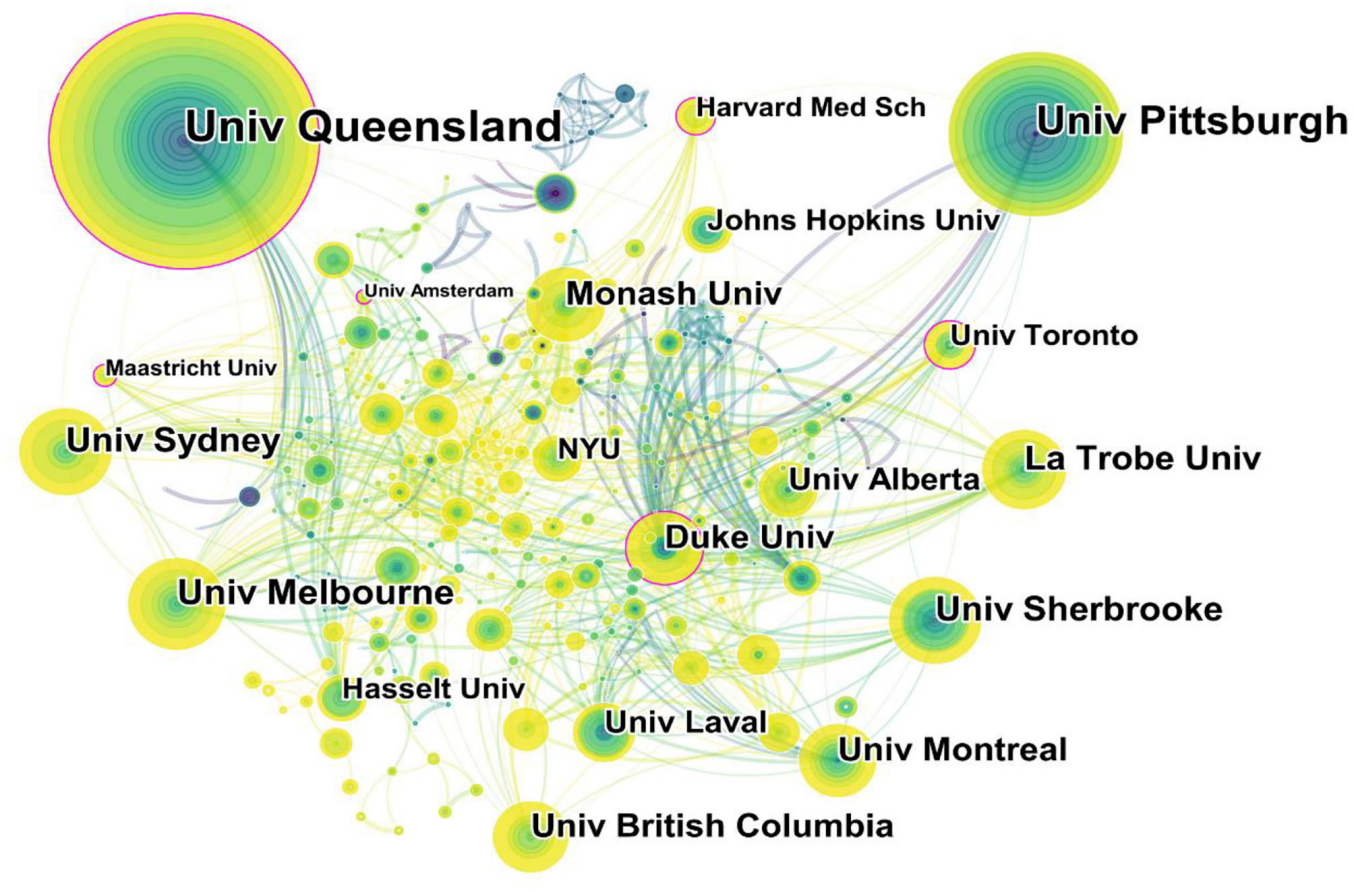
Map of institutions researching telerehabilitation from 2000 to 2021.

Of the 580 institutions which paid close notice in the field of telerehabilitation, the top 5 institutions were all universities (Figure 4). They were Univ Queensland, Univ Pittsburgh, Univ Melbourne, Univ Sherbrooke, and Univ Sydney. Moreover, the top five institutions in terms of centrality were Univ Maastricht (0.14), Univ Duke (0.12), Univ Amsterdam (0.12), Univ Toronto (0.11), and Harvard Med Sch (0.11). Analysis in terms of publication and centrality indicated that the main research institutions were Univ Queensland (Australia) and Duke Univ (United States). They were the cores that formed a complex cooperative network. Univ Queensland's research on telerehabilitation involved Parkinson's disease, heart failure, speech rehabilitation, and so on. The Duke Univ focused more on the telerehabilitation of stroke in the early years but shifted its focus to telerehabilitation of heart failure in recent years.

### Analysis of Authors and Cited Authors

**Figure 5:**
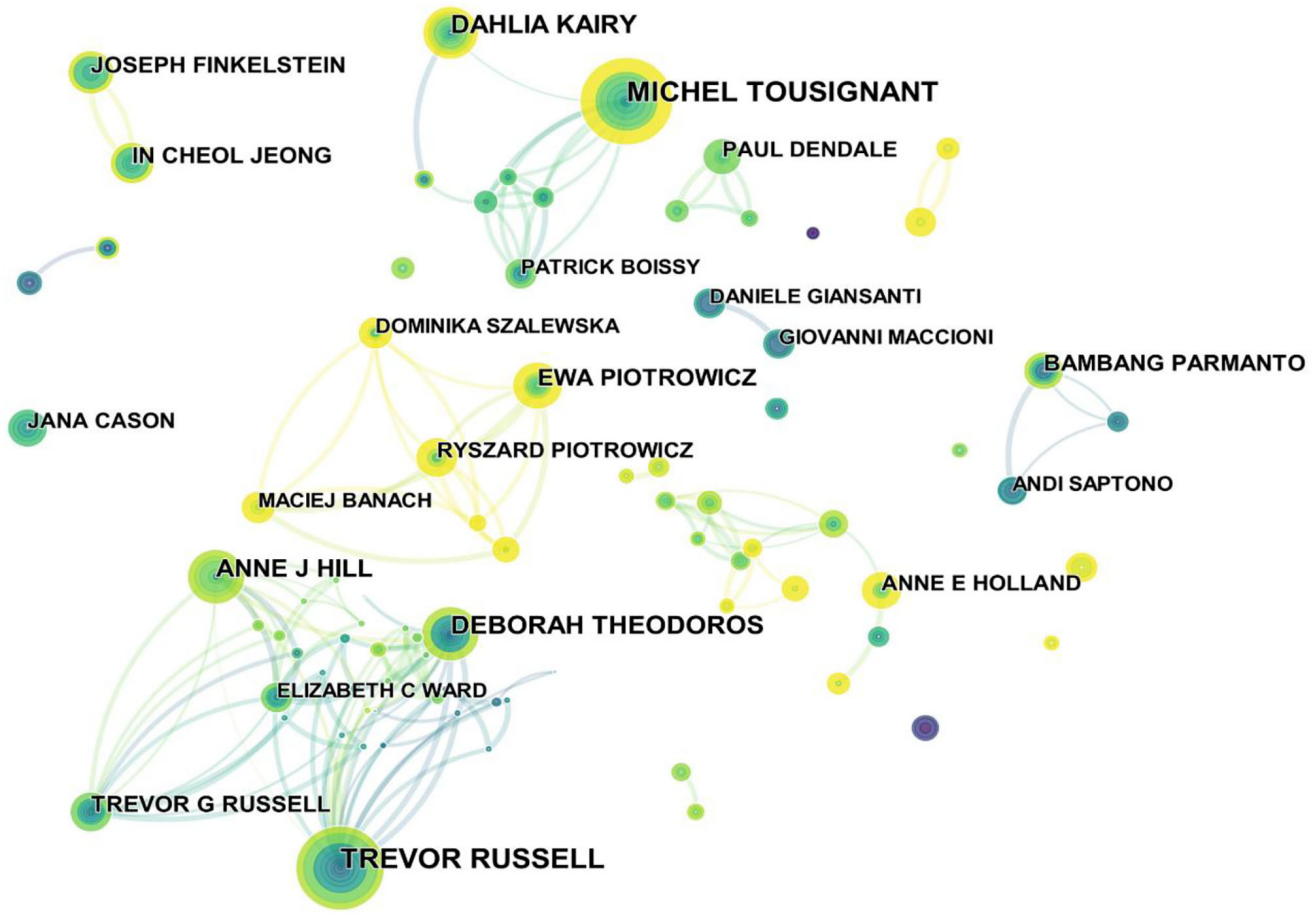
Map of authors related to telerehabilitation from 2000 to 2021.

The authors of the 1,986 publications were analyzed and resulted in 765 nodes and 1,285 links (Figure 5), indicating that the 1,986 articles were published by 765 authors. Among these authors, there were collaborative groups that formed a link with each other and scattered individual authors. The top five authors were Michel Tousignant (12), Trevor Russell (13), Deborah Theodoros (14), Dahlia Kairy (14), and Anne J Hill (15) in Figure 5. Michel Tousignant and Dahlia Kairy collaborated on telerehabilitation related to stroke and COVID-19 (16–18), in which they constructed a research idea of telebased tai chi exercise to intervene in the stroke population. In addition, they verified effective improvements in pulmonary symptoms and quality of life in seven COVID-19 discharged patients who received an 8-week exercise prescription for remote pulmonary rehabilitation, including respiratory training, cardiovascular exercises such as walking training, stair training, and resistance training. There were some collaborations between Trevor Russell, Deborah Theodoros, and Anne J Hill, and their collaboration paid more attention to speech disorders. They organized groups of aphasia patients to share their personal life histories *via* online video conferencing from their homes, which created opportunities for communicative success and built connections with others. Results showed that a multi-purpose group intervention for people with aphasia can result in improved communication, communicative participation, and quality of life (19). They also summarized the technical management review of communication and swallowing disorders in Parkinson's patients and found that the treatment of the speech disorder online was the most developed aspect of the technology-enabled management pathway (20).

**Figure 6:**
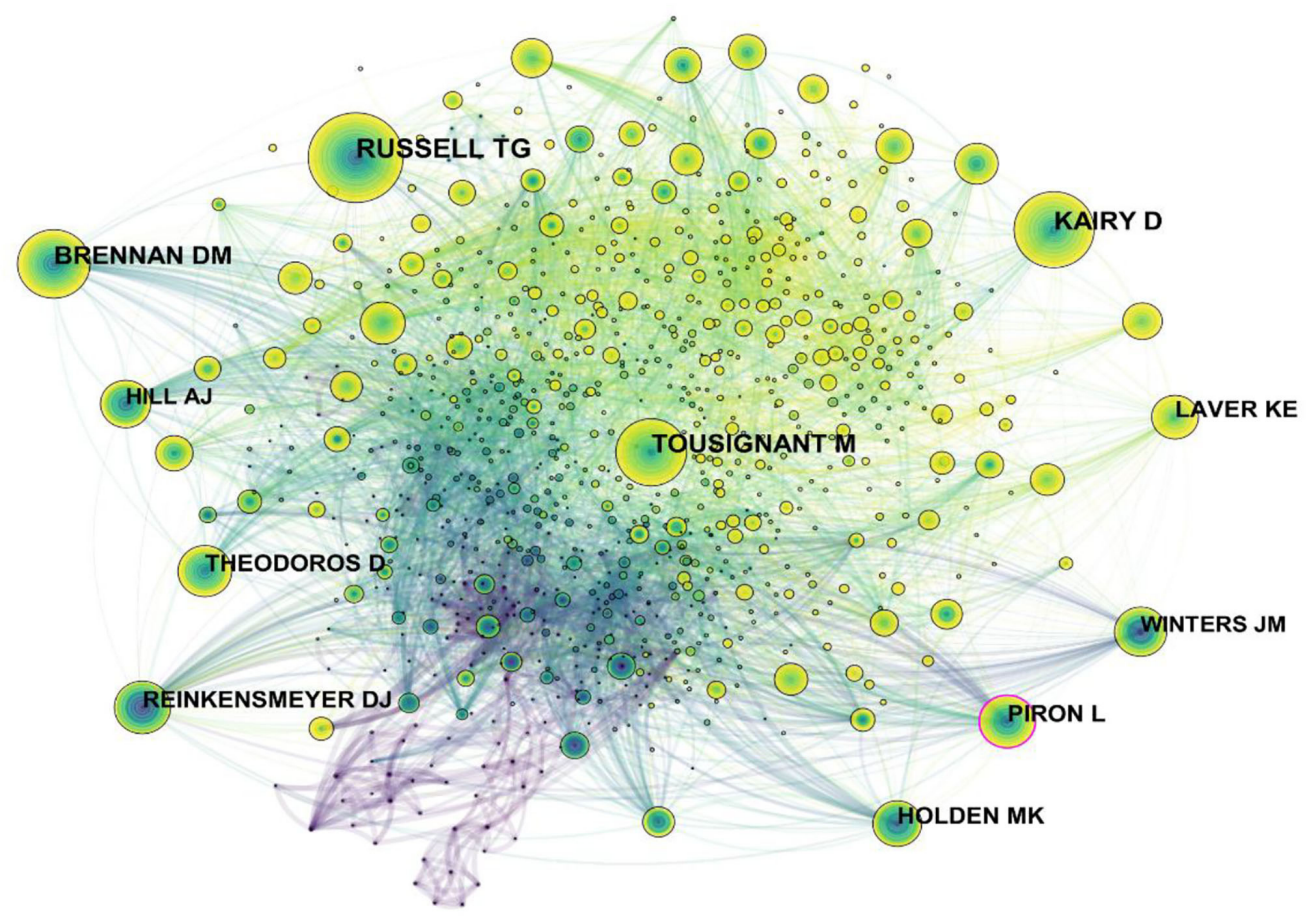
Map of cited authors related to telerehabilitation from 2000 to 2021.

The map of cited authors is displayed in Figure 6. Russell TG had the highest citation counts, followed by Kairy D, Tousignant M, Brennan DM, and Laver KE (Table 5). The top five authors in terms of centrality were Piron L, Tousignant M, Winters JM, Russell TG, and Hill AJ. In Figure 6, we can find that the node of “Piron L” had a distinct purple ring, indicating a high mediating effect, while the centrality of other nodes was not obvious. Piron L was based at the University of Padova (Italy) and had focused his vision on the combination of post-stroke rehabilitation with modern technology in the early 20th century. In 2001, he published a paper on the application of virtual reality as a tool to evaluate arm motor defects after brain lesions. The results showed a significant correlation with clinical scale scores (15). In 2009, PL combined telerehabilitation with virtual reality. A virtual reality system delivered over the internet that provides upper limb motor function training to stroke patients found that motor performance produced better results (14).

**Table 5:** Top five Frequency and centrality of cited authors related to telerehabilitation.

**Rank**	**Frequency**	**Author**	**Rank**	**Centrality**	**Author**
1	274	Russell TG	1	0.14	Piron L
2	219	Kairy D	2	0.09	Tousignant M
3	205	Tousignant M	3	0.09	Winters JM
4	175	Brennan DM	4	0.07	Russell TG
5	133	Laver KE	5	0.07	Hill AJ

Combining author, cited author, and centrality, Michel Tousignant (Tousignant M) was a professor in the field and had an important influence on the development of telerehabilitation. He was from the University of Sherbrooke (Canada), and he was mainly devoted to investigating the telerehabilitation of musculoskeletal conditions, especially after knee arthroplasty. One of his opinions was that home telerehabilitation and routine rehabilitation were not significantly different in therapeutic efficacy or satisfaction, and a favorable cost difference was observed when patients were more than 30 km away from the provider (21–23).

### Analysis of Cited References

**Figure 7:**
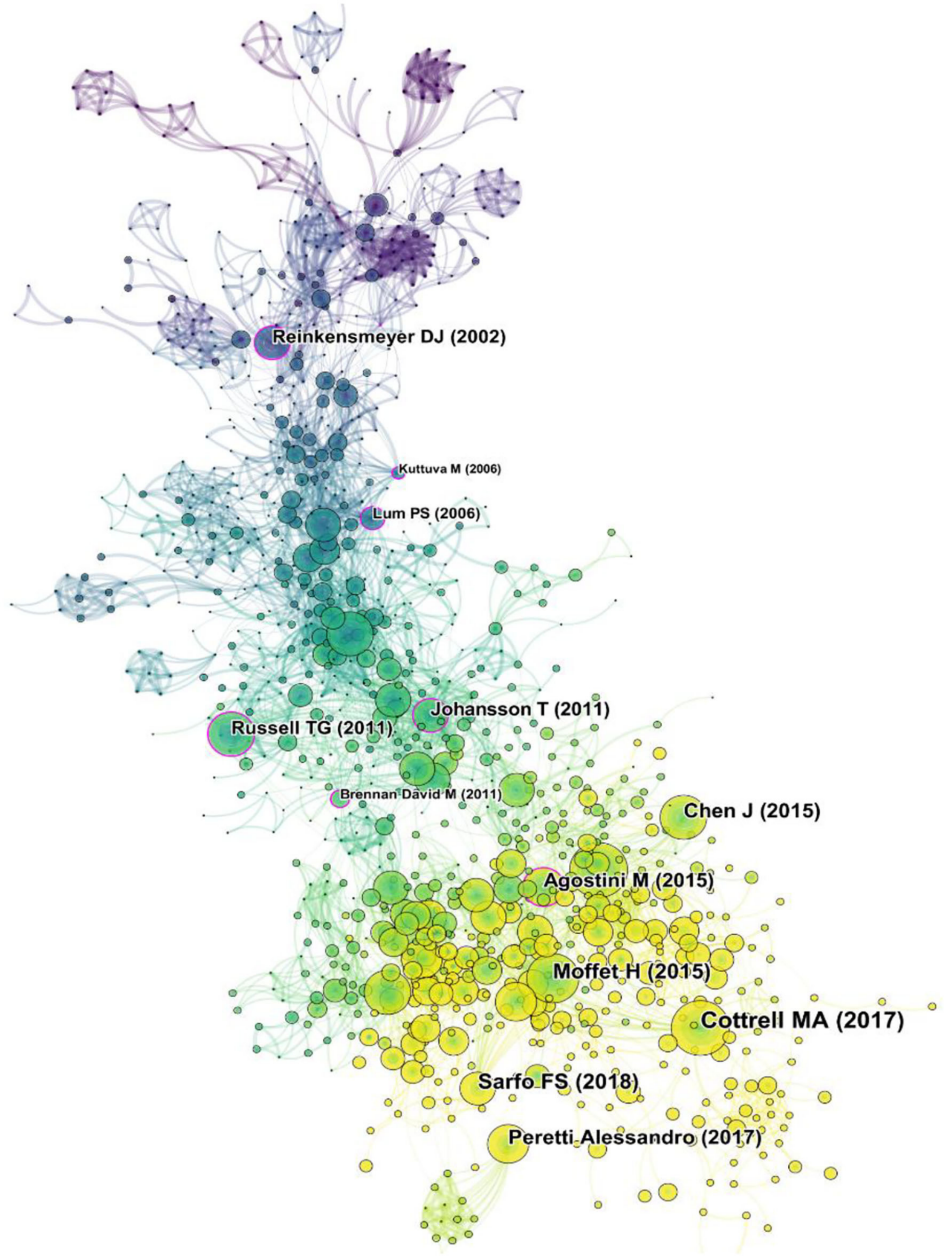
Map of cited references to telerehabilitation from 2000 to 2021.

Generating a cited reference co-citation map resulted in 1,141 nodes and 4,934 links (Figure 7). In terms of citation frequency, the first was the article published in 2017 by Cottrell MA (24). The article conducted the first systematic review to confirm the beneficial effect of real-time telerehabilitation for musculoskeletal conditions and suggested that rigorous clinical trials were warranted. Additionally, the randomized controlled trial published by Moffet H in 2015 (25), which was ranked the second list, demonstrated the non-inferiority of in-home telerehabilitation after total knee arthroplasty and supported its use as an effective alternative to face-to-face service delivery. The review study published by Peretti et al. (26), which ranked the fourth reference, made a starting point that improving approaches and devices for telerehabilitation emphasized the need for proper training and education of people involved in this new area. The third and fifth places (Sarfo FS, Chen J) were all systematic reviews about the telerehabilitation of stroke published in J Stroke Cerebrovasc Dis (13, 27).

The centrality of cite references ranked the first conducted by Russell et al. (1), who carried out a single-blinded, prospective, randomized, controlled non-inferiority trial to compare the equivalence of the internet-based telerehabilitation program and conventional outpatient physical therapy in the treatment of total knee arthroplasty and the results revealed that the efficacy of the two treatments was similar, but telerehabilitation had better performance in the stiffness subscale of the WOMAC and patient satisfaction. Lum PS developed a device called Automated Constraint-Induced Therapy Extension (“AutoCITE”) that automated the intensive training component of constraint-induced movement therapy to assess the effectiveness of “AutoCITE” training in a telerehabilitation setting when supervised remotely for participants with a chronic stroke (28). Brennan et al. (29), who was one of the members of the Telerehabilitation Special Interest Group, released the guideline about telerehabilitation in 2011 to inform and assist associated personnel in providing effective and safe services that were based on user needs, current empirical evidence, and valid technologies. The same centrality of Agostini M and Johansson T was published in J Telemed Telecare and IEE Trans Neural Syst Rehabil Eng, respectively (30, 31).

**Figure 8:**
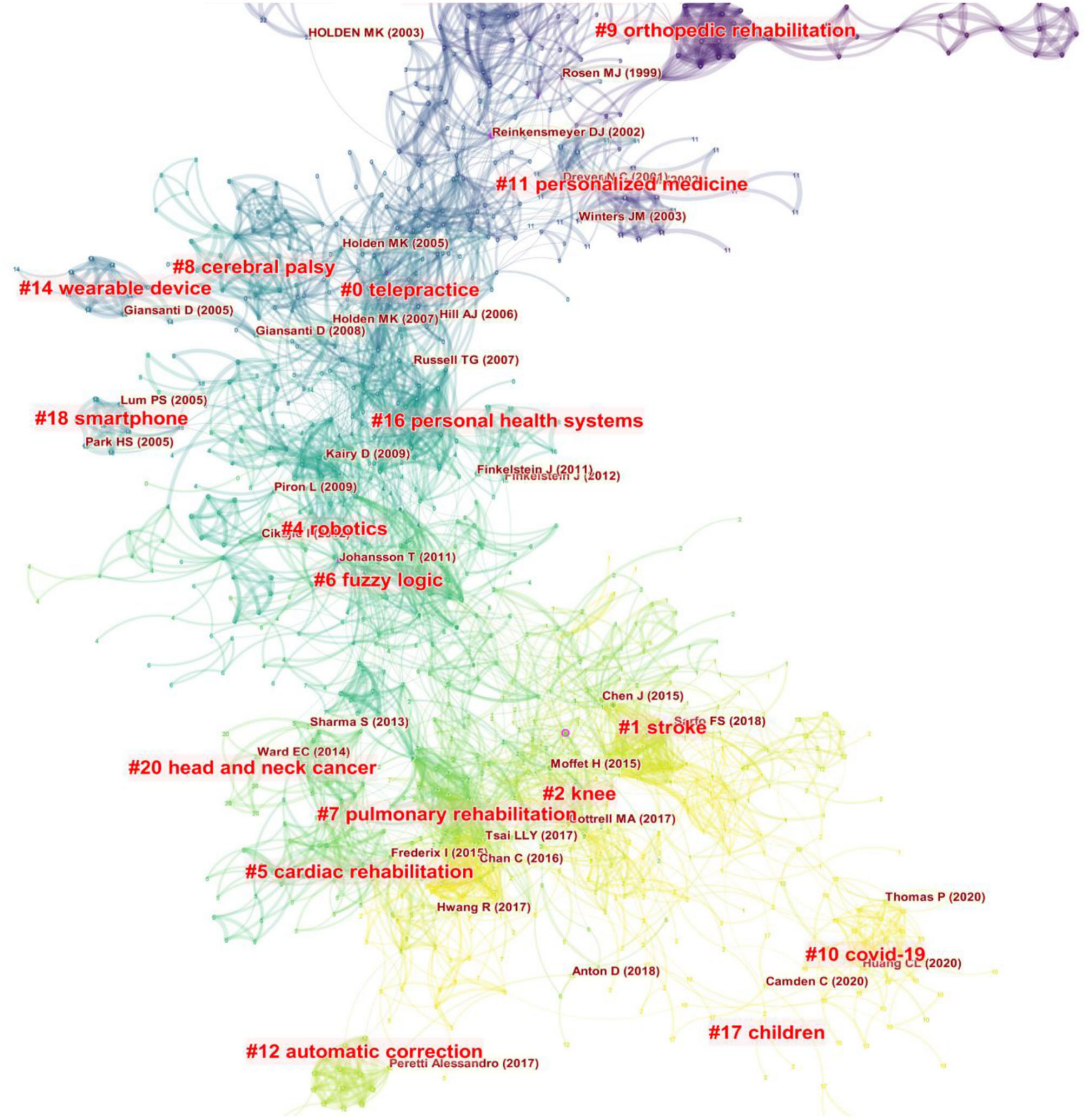
The clustering map of cited references related to telerehabilitation.

We performed cluster analysis on the cited references to clarify the topic and time distribution of these cited references (Figure 8). The Q value was 0.7984 and the S value was 0.9111 in this map, indicating that the clustering effect was good and the credibility was high. As can be seen from the colors in the figure, the most recent cited topics focus on stroke, knee, children, and cardiopulmonary rehabilitation. In addition to the above, another recent cluster is the outbreak of COVID-19 in recent years.

### Analysis of Keywords

**Figure 9:**
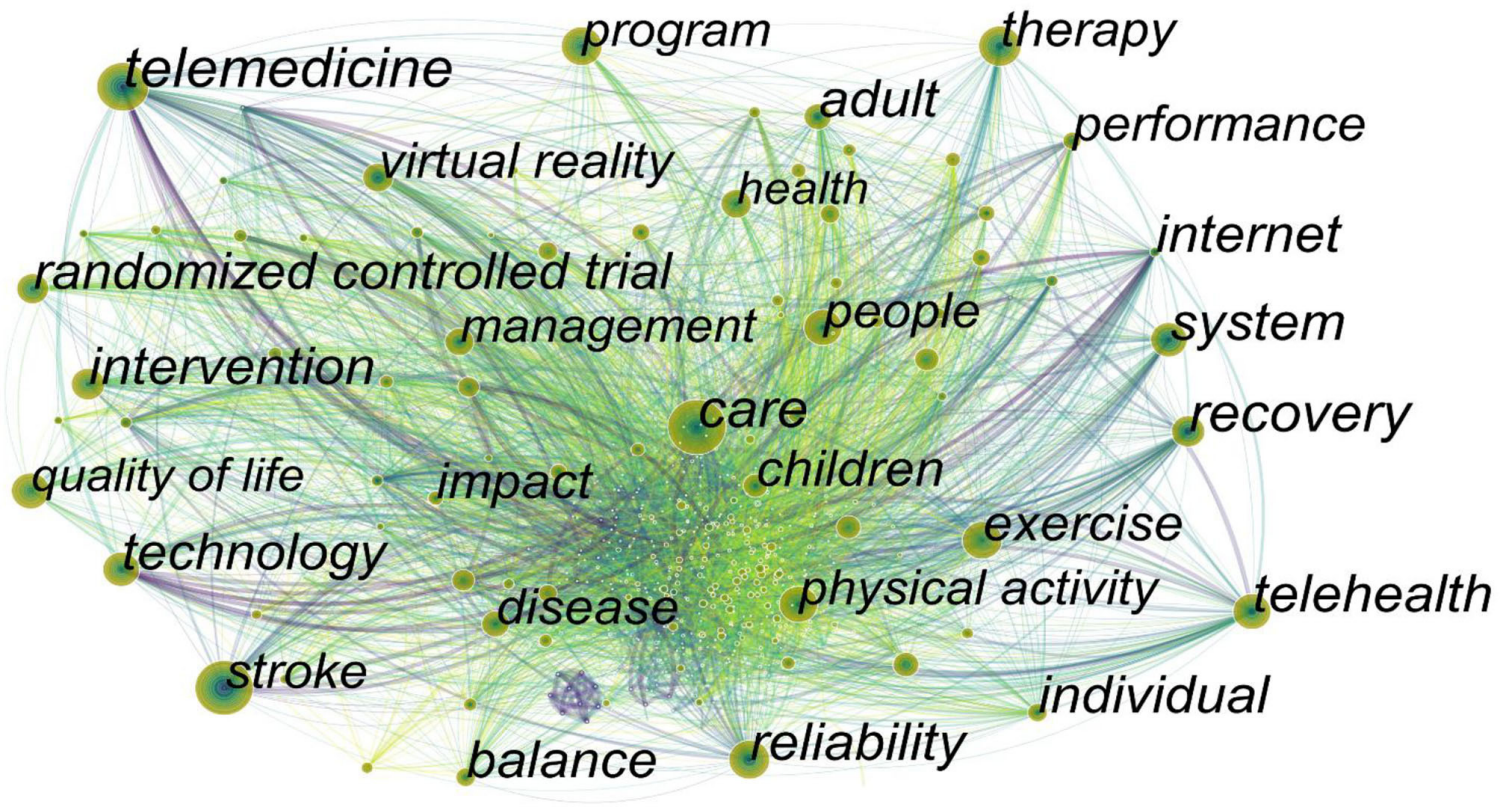
Map of keywords to telerehabilitation from 2000 to 2021.

It was considered that the indicators for evaluating the most leading-edge topics or emerging trends were the increased frequency of keywords or the increased number of keyword bursts in the citation within a certain period (32). The network map of keywords was generated and consisted of 541 nodes and 4,586 links (Figure 9). A total of 541 research keywords were identified in the field of telerehabilitation, which reveals the hottest topics. According to the frequency and centrality (Table 6), we can see the popular keywords were “care,” “stroke,” “telemedicine,” “exercise,” and “care,” which had a high frequency and centrality. As a modern technology, telerehabilitation reduces the pressure on carers and provides help for caring work at the same time. At present, the most concerned patients of telerehabilitation are stroke patients, who have a long course of the disease and require a certain amount of time to recover. However, the current hospital stay period is short and the cost is high. Telerehabilitation solves the problems above. It can not only help and supervise the rehabilitation of patients after discharge but also save costs and ease the burden on medical workers. “Exercise” is also a popular word. The biggest difference between telerehabilitation and face-to-face rehabilitation lies in the therapist's “hands-on” and “hands-off.” Therapists cannot touch patients with their hands, and the palpation and manipulation adjustments are limited in telerehabilitation. So, it is mostly about exercise instruction and training. Of course, exercise is also an important part of rehabilitation, which allows patients to participate actively, and remote management can better guide and supervise them. Furthermore, we can also find some words like “randomized controlled trial” and “quality of life” in the table. A randomized controlled trial is a gold standard for evaluating the efficacy of telerehabilitation, which provides high-quality evidence-based testimony. A single-blind method is used in most studies due to the particularity of intervention methods. The frequency of the keyword “quality of life” is increasing year by year, indicating that people pay much attention to the quality of life for patients after telerehabilitation and care about the physical and mental changes of patients. The main measurements include HRQoL, EuroQoL-5, and Short form-36 questionnaire score (33–35).

**Table 6:** Top 10 frequency and centrality of keywords to telerehabilitation.

**Rank**	**Keyword**	**Frequency**	**Rank**	**Keyword**	**Centrality**
1	Care	214	1	Care	0.08
2	Stroke	164	2	Recovery	0.07
3	Telemedicine	150	3	System	0.06
4	Exercise	136	4	Reliability	0.06
5	Program	136	5	Randomized controlled trial	0.06
6	People	133	6	Individual	0.06
7	Telehealth	132	7	Internet	0.06
8	Quality of life	127	8	Telehealth	0.05
9	Intervention	125	9	Intervention	0.05
10	System	120	10	Adult	0.05

**Figure 10:**
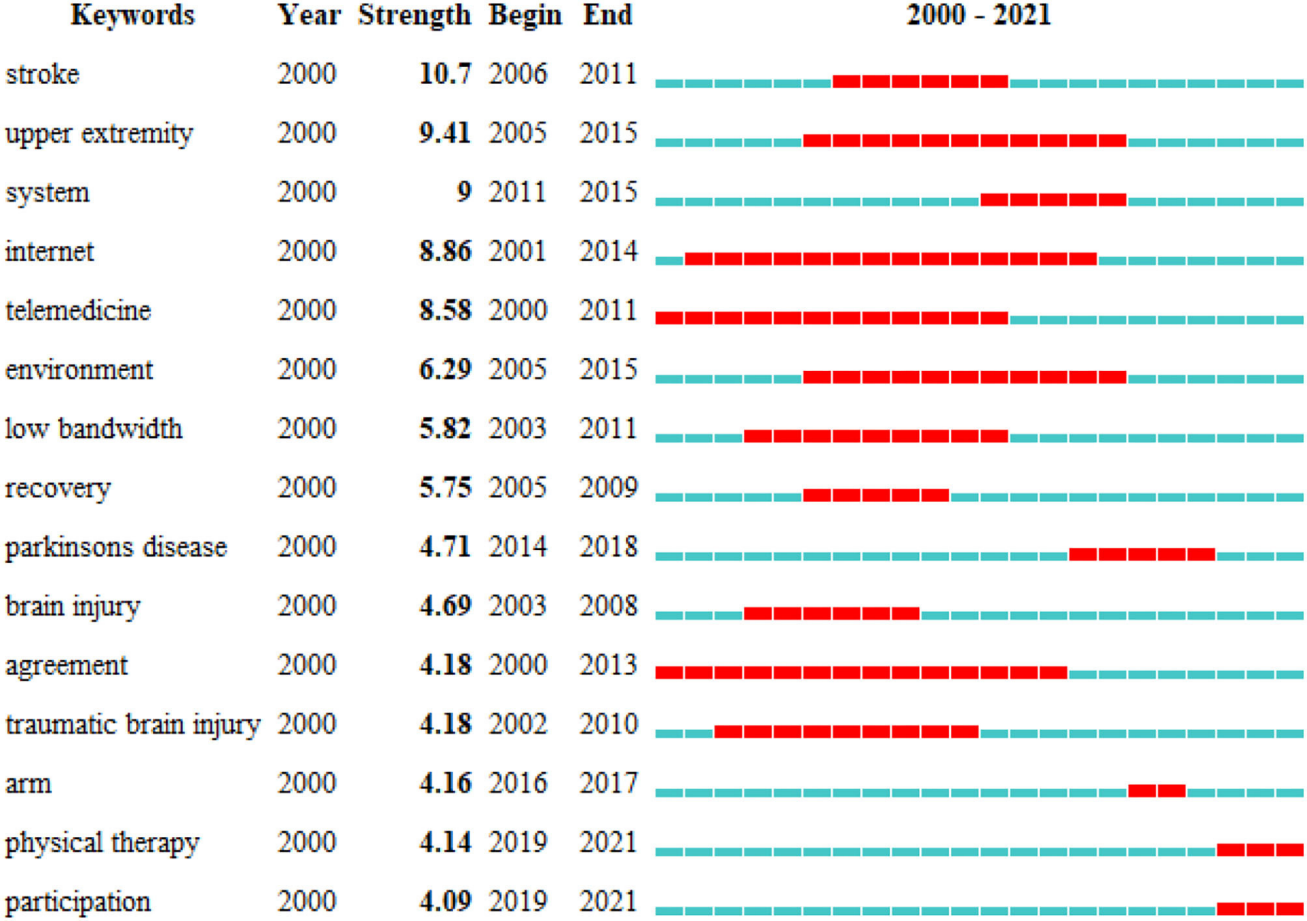
Top 15 keywords with the strongest citation burst.

The top 15 cited keywords with the strongest citation burst from 2000 to 2021 are shown in Figure 10. Among these keywords, “stroke,” “upper extremity,” “Parkinson's disease,” “brain injury,” “traumatic brain injury,” and “arm” are the objects of intervention for telerehabilitation. The keyword “stroke” emerging from 2006 had shown the strongest citation burst of 10.7 (164). The keywords such as “Internet,” “low bandwidth,” “system,” and “environment” were all about various technologies or application environments of telerehabilitation. Generally speaking, the technologies used for telerehabilitation can be divided into image-based telerehabilitation, sensor-based telerehabilitation, virtual environments, and virtual reality telerehabilitation (36). Videoconferencing is by far the most widely used, as per cost and technical difficulty. But back in the 1980s, telemedicine initially was limited to high-bandwidth applications. High cost, low access, and system complexity hindered the widespread adoption of broadband videoconferencing systems in medicine. Subsequently, successful research and application of low-bandwidth technologies enabled telemedicine to provide substantial help to underserved areas in a more affordable and accessible manner (37).

The most recent burst keywords were “physical therapy” and “participation.” Physical therapy, as a large part of rehabilitation, plays with the ability to develop, maintain, and rebuild movement and functional capacity. Telerehabilitation in physical therapy has gained a lot of attention and its application is gradually expanding. Future clinical trials should strictly consider internal validity and optimal sample sizes, and non-inferiority studies should be recommended to prove that telerehabilitation is not inferior to standard rehabilitation. Moreover, the challenge is the feasibility of telerehabilitation in a variety of resource settings (38). The keyword “participation” included the views of the implementor, participants, and their relatives on telerehabilitation, and the development of an ICT-based rehabilitation to support a person-centered rehabilitation process for survivors and their significant others. It establishes a sense of connection between the medical staff and the patient and reduces the patient's feeling of abandonment when rehabilitation ends (39). A study from Denmark examined the perspectives of physiotherapists and occupational therapists on ICT. They proposed to develop a diverse personalized app just for post-stroke survivors (40).

**Figure 11:**
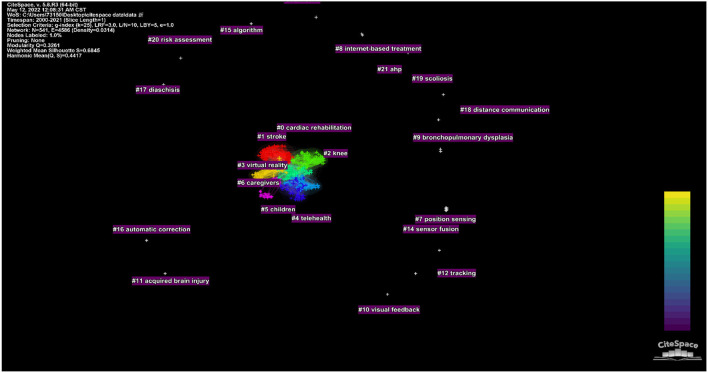
The clustering map of keywords related to telerehabilitation.

**Figure 12:**
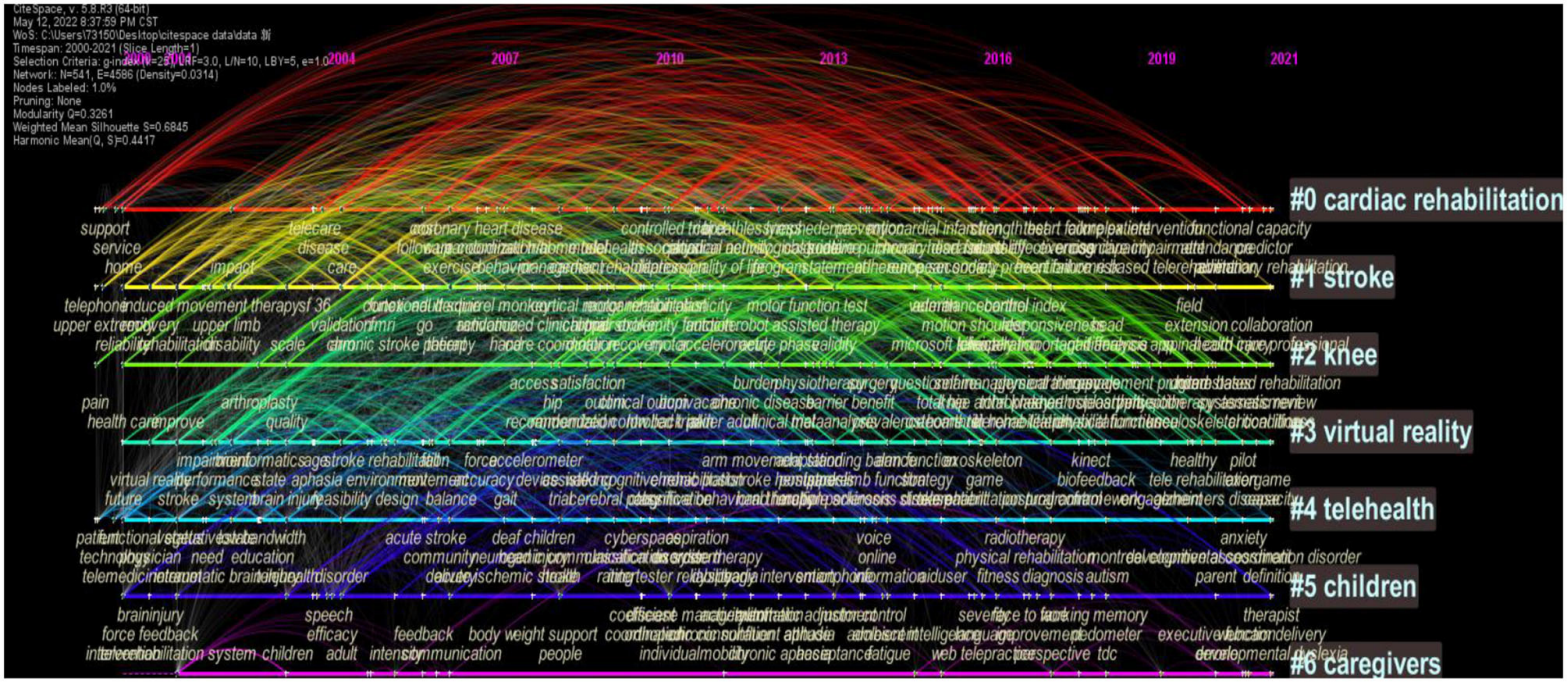
The timeline view of keywords related to telerehabilitation.

We conducted cluster analysis on these keywords and summarized them, so as to have a more intuitive understanding of the current research topics related to telerehabilitation. After clustering, the Q value is 0.4591 and the S value is 0.8497, indicating that clustering is appropriate and meaningful. A total of 21 clusters were generated to reflect the hot trends, among which the top six clusters containing the most keywords are “cardiac rehabilitation,” “stroke,” “knee,” “virtual reality,” “telehealth,” “children,” and “caregivers” (Figure 11). From the timeline view (Figure 12), in terms of color warmth, “stroke” and “cardiac rehabilitation” are the latest studies, while “knee” and “children” appear earlier. In addition, virtual reality technology is often involved in the field of remote rehabilitation. Many studies have found that remote integration with virtual reality technology can improve the motor function of the upper limb (41, 42). Virtual reality technology can improve the interest and enthusiasm for patient training, and their combination can play a greater therapeutic effect.

## Discussion

Telerehabilitation, as an emerging and alternative therapy, has been widely valued and studied with the gradual development of technology, especially during the epidemic period, and has the advantages of being cost-effective and convenient. In this study, we searched the core data of WOS based on the search formula and obtained 1,986 literature data on telerehabilitation research from 2000 to 2021. Based on the bibliometrics analysis of CiteSpace, the spatial and temporal distribution and research hotspots of telerehabilitation were clarified. Over the previous 20 years, the related publications increased at a rapid rate. Notably, the largest increase occurred between 2019 and 2020, which may be linked to the demand for remote technology during the spread of COVID-19. In this study, the Journal of Telemedicine and Telecare published the most articles (101) and was the biggest cited (997). The average IF, Eigenfactor, CiteScore, SNIP, and SJR of cited journals were 4.666, 0.0381, 5.750, 1.280, and 1.710, respectively. The countries and institutions that had issued studies in telerehabilitation had relatively close cooperation. Generally, the United States, Australia, and some other European countries, with a high publication rate and centrality, all developed countries, proved to be the main leaders under technological advantages in this field. The most productive institution was the University of Queensland in Australia. But an important trend that deserved attention was that the research achievements of Asian countries increased very fast after 2019, China was the most prolific country in the Asian region. A total of 765 active authors from various countries had attempted to estimate and evaluate the effectiveness of telerehabilitation for diseases such as stroke, knee arthroplasty, speech disorders, heart failure, and so on. In recent years, they have focused on telerehabilitation of lung function in a post-COVID-19 world and the applicability of telerehabilitation to other diseases during the COVID-19 pandemic. These authors performed randomized trials or systematic reviews to compare telerehabilitation with usual face-to-face rehabilitation. The results showed that the effect of telerehabilitation was as good as that of usual rehabilitation in a therapeutic effect, and it obtained the satisfaction of patients and their families. People from remote regions got higher benefits, mainly reflected in saving transportation costs and time. Among these authors, Tousignant M was the most productive author and Russell TG ranked the first among the cited authors. The first cited reference was a review on telerehabilitation of musculoskeletal diseases published by Cottrell MA in 2017, and a cluster analysis of cited references showed that stroke, cardiopulmonary rehabilitation, knee, children, and COVID-19 were the latest hot topics cited.

From the keywords, we found that “stroke” has been a hot topic of attention and research. For stroke patients, full recovery is not guaranteed after restorative rehabilitation in the acute and subacute stages of stroke. To maximize recovery and maintenance of function, patients with chronic stroke must undergo ongoing rehabilitation or exercise interventions. Telerehabilitation helps stroke patients maintain vertical continuity of motor rehabilitation at home and helps reduce the workload of therapists. Therefore, stroke is in great demand for telerehabilitation and it has always been a research hotspot. In addition, cardiac rehabilitation, knee rehabilitation, and child rehabilitation are also the main application fields of telerehabilitation. Developments and innovations in telerehabilitation technology are being studied extensively. At present, video conferencing is the most widely used, and internet-based low-bandwidth communication provides a more cost-effective and easily accessible telemedicine solution. With the continuous development of modern technology, the combination of virtual reality technology and telerehabilitation brings new opportunities and development space. The frontier keywords were “physical therapy” and “participation”. Since the COVID-19 outbreak, the World Confederation for Physical Therapy's task force has proposed a pragmatic approach to shifting service paradigms and scaling up telehealth physical therapy within a large medical center (43). In the future, higher quality clinical trials and systematic reviews are imperative in this important field of investigation. In addition, telerehabilitation aims to benefit people, so individualized intervention programs should be made based on the situation of different patients. Satisfaction surveys should not only be conducted for patients but also the opinions of family members and therapists should be included. Telerehabilitation development needs to support person-centered concepts in the future.

## Limitation

Several limitations need to be noted regarding this study. First, we only analyzed the data from the Web of Science. Therefore, the results may not be comprehensive, and it is necessary to combine more database resources for analysis in the future. Second, although the search terms chosen were considered, we cannot guarantee that every piece of literature is completely related to the topic, and it is also uncertain whether all documents related to the topic have been retrieved. Third, if the search is carried out at a different period, the citation counts and centrality of the articles may be different. So, this study only represents the research in the past 20 years. It is necessary to update the study in the future. Even so, we believe that this study can still be used to describe the overall situation and developing trends in this field from 2000 to 2021 and provide suggestions for follow-up research.

## Conclusion

In summary, this study uses visualization software CiteSpace to identify new perspectives concerning potential collaborators and cooperative institutions, hot topics, and research frontiers in the research field, providing a direction for exploring and developing telerehabilitation. Telerehabilitation research is mostly done in developed countries, and cooperative networks have been formed among the authors. Randomized controlled trials and reviews prove that telerehabilitation and traditional face-to-face rehabilitation are equal but more cost-effective. At present, the main area of concern for telerehabilitation is stroke, and future research may turn to the applicability of telerehabilitation in physical therapy in the pandemic era. At the same time, remote technology will be further developed in the future, allowing people in remote areas to access low-cost and stable internet configurations. In addition, the combination of telerehabilitation and virtual reality technology is also a highlight. In the future, the diagnosis and treatment of telerehabilitation will pay more attention to the people-centered concept and develop personalized diagnosis and treatment plans, which will not only satisfy patients but also be recognized by family members and therapists. With the continuous spread of the pandemic, telerehabilitation will be further studied and promoted.

## Data Availability Statement

The original contributions presented in the study are included in the article/supplementary material, further inquiries can be directed to the corresponding author.

## Author Contributions

MH contributed to conceive, design, and revise manuscript. JZ and LL contributed to data collection and analysis and manuscript writing. XW contributed to obtaining funding for the study and manuscript revision. All authors contributed to the article and approved the submitted version.

## Funding

This work was supported by the National Natural Science Foundation of China (Grant nos. 81774384 and 82074515) and the National Joint Engineering Research Centre of Rehabilitation Medicine Technology (Grant no. X2018002-platform).

## Conflict of Interest

The authors declare that the research was conducted in the absence of any commercial or financial relationships that could be construed as a potential conflict of interest.

## Publisher's Note

All claims expressed in this article are solely those of the authors and do not necessarily represent those of their affiliated organizations, or those of the publisher, the editors and the reviewers. Any product that may be evaluated in this article, or claim that may be made by its manufacturer, is not guaranteed or endorsed by the publisher.
